# Curated Collections for Educators: Five Key Papers about Residents as Teachers Curriculum Development

**DOI:** 10.7759/cureus.2154

**Published:** 2018-02-04

**Authors:** Sara M Krzyzaniak, Alan Cherney, Anne Messman, Sreeja Natesan, Michael Overbeck, Benjamin Schnapp, Megan Boysen-Osborn

**Affiliations:** 1 Emergency Medicine, University of Illinois College of Medicine at Peoria/osf Healthcare Saint Francis Medical Center; 2 Emergency Medicine, Thomas Jefferson University Hospitals; 3 Emergency Medicine, Wayne State University; 4 Emergency Medicine, Duke University Medical Center; 5 Emergency Medicine, University of Colorado School of Medicine; 6 Emergency Medicine, University of Wisconsin; 7 Emergency Medicine, University of California at Irvine

**Keywords:** medical education, curriculum development, program evaluation, residents as teachers, curated collection, modified delphi method

## Abstract

The Accreditation Council for Graduate Medical Education (ACGME) requires residency programs to prepare residents to teach and assess medical students and other learners. In order to achieve this, many programs develop formal residents as teachers (RAT) curricula. Medical educators may seek the guidance of previously published literature during the development of RAT programs at their institutions.

The authors sought to identify key articles published on the subject of RAT programs over the last 10 years. The authors utilized a formal literature search with the help of a medical librarian and identified additional articles from virtual discussions among the author group and an open call for articles on Twitter using the hashtag #MedEd. Virtual discussions occurred within an online community of practice, the Academic Life in Emergency Medicine (ALiEM) Faculty Incubator. The lead author conducted a four-round modified Delphi process among the author group in order to narrow the broad article list to five key articles on RAT programs. The authors summarize each article and provide considerations for junior faculty as well as faculty developers.

Curriculum development and program evaluation should utilize established frameworks and evidence-based approaches. The papers identified by this Delphi process will help faculty use best practices when creating or revising new RAT curriculum. In addition, faculty tasked with guiding junior faculty in this process or creating faculty development programs around curriculum development will find these articles to be a great resource for building content.

## Introduction and background

The Accreditation Council for Graduate Medical Education (ACGME) expects residents to develop the skills necessary to teach residents, medical students, patients, families, and other health professionals [1]. Similarly, the Liaison Committee on Medical Education (LCME) requires medical schools to train residents in teaching and assessment [2]. Residents commonly provide clinical teaching for other learners; in fact, resident teaching time may exceed that of attending physicians, with up to one-third of student learning coming from residents [3,4]. Previous studies have shown that residents are an effective supplement to faculty [5]. Residents also report the desire to improve their teaching skills [6].

Similar to faculty development programs, residents as teachers (RAT) programs have a breadth of topics they may cover. A needs assessment of emergency medicine program directors found that feedback, communication and presentation skills, case-based teaching, bedside teaching, small group teaching, procedural skills teaching, assessment and evaluation, and teaching/learning styles were subjects covered in over half of existing RAT programs [7]. Ironically, lecture was the most commonly used instructional modality [7].

Despite the importance of developing teaching skills in residents, many programs do not have a formal RAT or near-peer educator curriculum [7–9]. Among programs with an established curriculum, there is little standardization [9,10]. Residents often report improved attitudes towards teaching and increased confidence in their teaching skills following RAT courses [11,12]; however, few program directors consider their RAT curricula to be very effective [13].

Academic faculty may be tasked with creating or evaluating curricula for local, regional, or national RAT programs. While it is essential that residents develop teaching skills during residency [1], there is little guidance for faculty on how to create a RAT program. The objective of this narrative review is to highlight key literature to guide faculty as they seek to develop, implement, and evaluate RAT curricula.

## Review

Methods

The Academic Life in Emergency Medicine (ALiEM) Faculty Incubator is an online community of practice for medical educators. A subgroup of participants from the 2017-2018 cohort was chosen to evaluate the literature surrounding RAT program development, based on an expressed interest in this topic and previous experience with RAT curricula. This group was composed of three faculty mentors and four junior faculty mentees.

**Figure 1:**
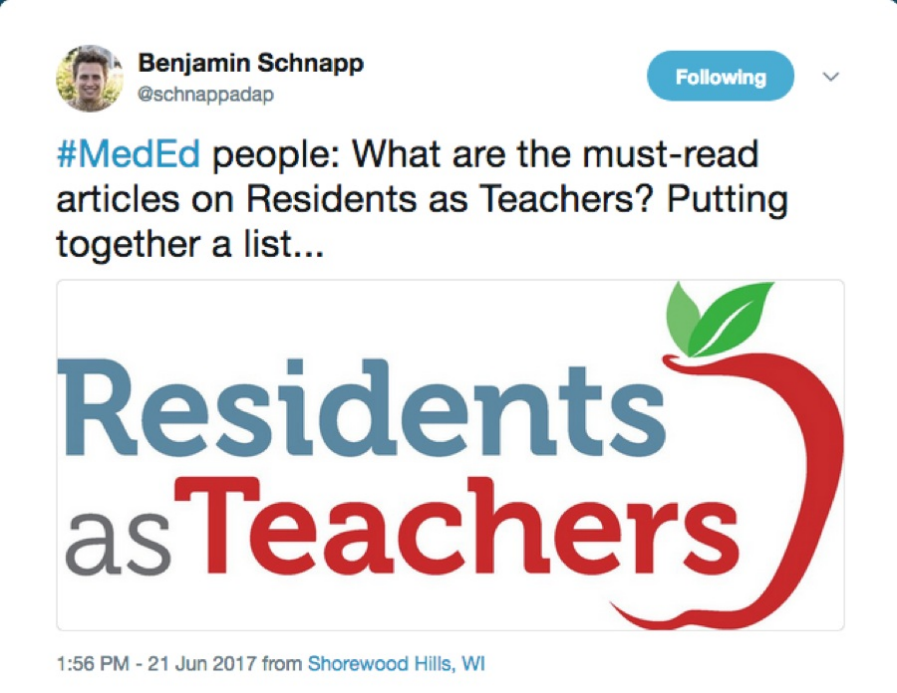
Tweet by Benjamin Schnapp soliciting requests for key papers on the topic of residents as teachers.

We performed a literature search with the help of a medical librarian. We gathered additional articles from bibliography reviews of previously identified articles, virtual discussion among our group, and through an open call on Twitter with the hashtag #MedEd (Figure 1). In order to focus on programs specific to residents, we excluded articles that only developed the teaching skills of faculty or residents (e.g., faculty development programs). In recognition of the changes to health care delivery and medical education in the last 10 years, we excluded articles that had been published more than 10 years ago.

This paper intends to identify core articles that may be used by junior faculty tasked with developing a RAT program at their institution and/or faculty developers who guide junior faculty through the curriculum design process.

The final group of five papers was identified through a four-round voting process akin to the Delphi methodology, utilized previously in key articles [14-24]. Each round of voting was completed electronically using Google Forms. The participants ranged from junior to senior faculty who had existing experience and interest in RAT curriculum development. Every participant read the initial group of 24 articles and voted during each round.

In the first round, raters were instructed to indicate the importance of each article using a seven-point Likert scale. The lowest option (one) was anchored by the statement “unimportant for junior faculty” and the highest option (seven) was anchored by the statement “essential for junior faculty.” During the second round, raters were provided with a frequency histogram displaying how each article had been rated in the previous round. Participants were then asked to indicate if each article “must be included in the top papers” or “should not be included in the top papers.” In the third round, raters were provided with the results of the second round as the percentage of raters who indicated that each article must be included. Participants were subsequently instructed to select the five papers that were most important for inclusion in the article. A fourth-round was required to break a tie between three papers with equal scores in the third round. The ALiEM Faculty Incubator used similar methods in an earlier series of papers published in the Western Journal of Emergency Medicine and Population Health [15-22] and Cureus [23,24].

Results

Our literature search and social media calls yielded 24 articles or book chapters published in the last 10 years on the topic of RAT curricula (Table 1). The four-round modified Delphi process allowed our team to generate a list of the papers perceived to be most relevant for faculty members tasked with developing and evaluating RAT curricula. A fourth-round was required to break a tie between three papers equally endorsed in the third round. The top five papers are further discussed below. Table 1 lists each paper with its citation followed by the rating it received in each round.

**Table 1:** The complete list of residents-as-teachers literature reviewed by authorship team.

Citation	Round 1: Initial Mean Scores (SD) Max score 7	Round 2: % of raters that endorsed this paper	Round 3: % of raters that endorsed this paper in last round	Round 4: Tie break round	Top five papers
Ramani S, et al. [25]	5.6 (1.3)	100%	85.7%		1st
Bree KK, et al.[8]	5.7 (1.1)	100%	71.4%		2nd
Chokshi BD, et al.[11]	5.6 (1.1)	57.1%	28.6%		
Ahn J, et al. [7]	5.4 (1.7)	71.4%	57.1%	33.3%	
Coverdale JH, et al.[26]	5.4 (1.7)	50%	28.6%		
Chisholm CD [27]	5.3 (1.8)	42.9%	28.6%		
Post RE, et al.[28]	5.3 (1.7)	85.7%	71.4%		3rd
Mann KV, et al.[29]	5.1 (2.0)	71.4%	57.1%	83.3%	4th (tie)
Ostapchuk M, et al.[30]	5.1 (1.9)	71.4%	57.1%	83.3%	4th (tie)
Sherbino J, et al.[31]	5.1 (1.6)	57.1%	14.3%		
Ilgen JS, et al. [32]	5.0 (1.9)	57.1%	0%		
Butani L, et al. [33]	4.9 (1.5)	57.1%	0%		
Hill AG, et al. [10]	4.4 (2.2)	28.6%	14.3%		
Miloslavsky EM, et al. [34]	4.0 (1.4)	28.6%	0%		
Smith CC, et al. [35]	4.0 (1.4)	0%	0%		
Hosein Nejad H, et al. [36]	3.7 (1.4)	0%	0%		
Jibson MD, et al. [37]	3.5 (2.1)	0%	14.3%		
Lacasse M, Ratnapalan S [38]	3.4 (1.4)	0%	0%		
Kensinger CD, et al. [39]	3.1 (2.0)	0%	0%		
Polan HJ [40]	3.0 (1.4)	0%	0%		
Soriano RP, et al. [41]	3.0 (1.4)	0%	0%		
Aba Alkhail B [42]	2.9 (1.2)	0%	0%		
Grady-Weliky TA, et al. [43]	2.4 (1.3)	0%	0%		
Halestrap P, Leeder D. [44]	2.1 (1.1)	0%	0%		

Discussion

The following is an annotated bibliography of five articles determined to be the most relevant for faculty members tasked with developing and evaluating RAT programs. Each article has a summary followed by a description of relevance for junior faculty members as well as the importance for senior faculty members when using these articles for faculty development workshops or sessions.

1. Mann KV, Sutton E, Frank B. Twelve tips for preparing residents as teachers. Med Teach. 2007;29:301-306 [29].

Summary

Drawing from their experience in creating a multidisciplinary four-week RAT elective, the authors offer 12 steps for implementing a successful RAT program. Planning for a RAT program should involve identifying specific institutional needs, building partnerships with educators and administrators, planning the goals and structure, determining the content, and choosing teaching and learning methods. The implementation phase should include authentic teaching practice, building residents’ awareness of themselves as teachers, planning for and anticipating challenges, and gathering regular feedback from the program’s participants. After the program is complete, it is important to evaluate the program and provide support for the participants to incorporate the program’s objectives into their daily teaching practices.

Relevance to Junior Faculty Members

This article provides a framework for developing a RAT curriculum that junior faculty may use when developing similar courses at their institutions; in fact, the authors offer readers the opportunity to communicate directly with them for more specific details of the curriculum.

Many of the pearls provided by the article may help junior faculty to implement successful RAT programs while avoiding pitfalls. The authors suggest matching the program’s content with targeted needs of the institution. For example, if the undergraduate medical education curriculum uses problem-based learning (PBL) heavily, then the same institution’s RAT curriculum should cover PBL. The 12 steps also emphasize the importance of follow-up, re-assessment, and feedback for both the participants and the faculty who are running the course.

Junior faculty members must balance the authors’ “tips” with the realization that the authors do not provide quantitative data for the effectiveness of their program. They rely on their own experience with a single, albeit multidisciplinary, program.

Considerations for Faculty Developers

While the authors do not explicitly cite Kern [45], the tips closely follow his six-step approach to curriculum development. The article offers practical ways to implement each step of curriculum design, specific to a RAT program. Furthermore, many of the steps offered by this article are relevant to faculty developers planning faculty programs, not just resident programs.

2. Ostapchuk M, Patel PD, Hughes Miller K, Ziegler CH, Greenberg RB, Haynes G. Improving residents’ teaching skills: A program evaluation of residents as teachers course. Med Teach. 2010;32:e49-e56 [30].

Summary

Prompted by an LCME citation, the authors describe the implementation of a campus-wide RAT curriculum at the University of Louisville School of Medicine, based on the University of California Irvine’s “Bringing Education and Service Together” (BEST) model [46]. Interns from all specialties attended off-site, daylong workshops during which they were protected from clinical duties. Small groups consisted of seven to nine participants, led by two volunteer faculty who had previously received instruction in the BEST model. Topics included microskills of teaching, orienting learners, giving feedback, teaching procedures, bedside teaching, and lecture skills. Impact of the program was based on post-session learner questionnaires, facilitator questionnaires, clerkship evaluations by third-year medical students, learner focus group feedback, and anecdotal reports of the perception of the intervention. The authors attempted to frame the impact of the curriculum in terms of Kirkpatrick’s Model and presented reasonable evidence of Level 1 and 2 achievements by participant residents [47]. Beyond attitudinal responses of residents involved in the program, the authors evaluated the impact of the program on third-year medical students through open-ended questions in focus groups, demonstrating that students identify and value several skills contained in the RAT curriculum: demonstrating respect, finding the time to teach, listening, and contextualizing teaching to current clinical cases. Continued measurement of the impact of resident instruction on the teaching of medical students is planned and should lend support to the assertion that residents are progressing up Kirkpatrick’s outcome hierarchy.

Relevance to Junior Faculty Members

The RAT curriculum presented by Ostapchuk, et al., is based on a successful curriculum originally developed to provide teaching skills to primary care residents [46]. This report highlights one approach to implementing RAT curricula (a concentrated daylong workshop) with good detail about topics covered, the need for curriculum revision year over year, and methods to measure the impact of the program. The workshops focused on six topics which were covered in a single day ‘retreat.’ Each topic was delivered using mini lectures, small group discussion and practice with standardized learners. Methods for evaluating the effectiveness of the curriculum included post-session questionnaires of participants and facilitators, as well as questionnaires and focus group interviews of third-year medical students specifically regarding residents as teachers. The important context for this curricular implementation is the LCME’s identification of deficient surgical resident instruction of medical students, and this is where most improvements were seen.

Considerations for Faculty Developers

Two important points stand out in reviewing Ostapchuk, et al.’s program. First, this article describes a curricular response to an identified weakness in teaching during an LCME site visit. The article forms a basis for the institution’s response to the citation, but also provides a blueprint for implementing a RAT curriculum modeled on the BEST model as originally described by Morrison, et al. [46]. Ostapchuk, et al.’s program relied on recruited faculty volunteers who underwent instruction on the BEST model by the Assistant Dean for Graduate Medical Education. The details of faculty development are missing. The training of faculty delivering content in Morrison’s original program is likewise vague, characterized as “generalist attending physicians with experience teaching faculty how to teach.” Thus implementing a RAT curriculum in the manner of Ostapchuk leaves the aspect of faculty development to deliver content and moderate discussions up to you.

Second, Ostapchuk, et al. continue the time-honored struggle to ascend Kirkpatrick’s hierarchy [47] in the evaluation of educational interventions. While reaction and learning are reflected in the attitudinal survey results of all involved, Ostapchuk, et al. move a step further in elaborating some effect on the long-term instructional impact on the target group: third-year medical students on a surgery clerkship. Through both a school-wide questionnaire as well as focus group interviews of third-year students, the authors demonstrate improvement in student perceptions of resident teaching over the study period, intimating achievement of Kirkpatrick’s Level 3 and 4 (behavior change and results).

3. Ramani S, Mann K, Taylor D, Thampy H. Residents as teachers: Near peer learning in clinical work settings: AMEE Guide No 106. Med Teach. 2016;38:642-655 [25].

Summary

This article provides a theoretical grounding for each step of RAT curriculum development using the Dundee 3-circle model, the Kellogg Program Logic model and the Kirkpatrick model for program evaluation. The first portion of the article discusses why the effort to create a RAT curriculum is worthwhile, showing impact at each level of Kirkpatrick’s model, from Reaction to Results as well as benefits for junior learners, residents, and the institution. The second section discusses the ideal design of a RAT curriculum, using the Kellogg Program Logic model to move from a needs assessment through learning outcomes and timelines to program evaluation, offering practical tips along the way to demonstrate the applicability of each stage of the model. The third section covers the ongoing maintenance of a RAT program by using the Dundee 3-circle model, ensuring program quality by verifying that the ‘right thing’ is being ‘done right’ by the ‘right person’ through tools such as reflective practice, role-modeling, and faculty development. Finally, the article concludes with a discussion of strategies to aid in successful implementation of a program customized to the needs of a local institution as well as potential pitfalls that have interfered with RAT initiatives in other environments.

Relevance to Junior Faculty Members

While it may be intuitively obvious to education-minded faculty that RAT programs and content are worthwhile, faculty members trained under a more traditional medical education paradigm may not share this perspective. Being able to offer empiric evidence of the effectiveness of RAT programs at other institutions for multiple stakeholders, including students, residents, faculty and the department may be effective at increasing momentum for a RAT curriculum. The idea of developing and implementing an entire RAT program can also seem overwhelming to a junior faculty member tasked with such a project. By placing RAT programs into a broader, context of widely accepted educational theoretical frameworks, junior faculty may be able to more easily understand the component parts of such a program as well as how and why to execute them systematically.

Considerations for Faculty Developers

Faculty developers must be well-versed in education theories and conceptual frameworks relevant to curriculum development and program evaluation. The article offers three robust frameworks to consider when developing a RAT curriculum. The Dundee 3-circle model guides content development, the Program Logic model frames curriculum development, and the Kirkpatrick model directs program evaluation. While not a prescriptive ‘best practices’ article per se, the article uses these frameworks to guide program development based on the resources and needs at one’s local institution. The article offers a solid conceptual backbone to help guide the mentorship of faculty who are developing RAT programs. It further delineates the faculty support necessary to develop such a program. Alternatively, for programs that may be encountering difficulties with support for their RAT curriculum, this richly referenced article may be more valuable as a persuasive tool, offering solid evidence and context within established educational theory of the value of a properly executed RAT program.

4. Post RE, Quattlebaum RG, Benich JJ. Residents-as-teachers curricula: A critical review. Acad Med. 2009;84:374-380 [28].

Summary

This paper provides firm recommendations on how residents should be instructed to teach and how a RAT curriculum is best evaluated.

The authors performed a comprehensive literature search, examining articles pertaining to RAT curricula from 1975-2008. Ultimately, they found 24 articles that provided both a description of the RAT curriculum and the subsequent program evaluation methods. The authors provide descriptions of these curricula and how their effectiveness was evaluated. They also provided brief appraisals of each of the curricula. Based on these 24 articles, the authors make a specific evidence-based recommendation, specifically that the One-Minute Preceptor [48] should be used as the foundation for RAT curricula. The effectiveness of RAT curricula should be evaluated through a randomized controlled trial (RCT) using Objective Structured Teaching Examinations (OSTEs) performed before and after the intervention. More specifically, they recommend that the RAT curricula last at least three hours with periodic reinforcement. When constructing one’s learner cohort to evaluate outcomes, the authors recommend at least 40 residents of varying post-graduate levels to provide adequate power for the program evaluation. They also recommend repeating the OSTE in six months-one year to assess long-term curriculum effectiveness.

Relevance to Junior Faculty Members

The article provides the reader with a summary of 24 articles published over a 33-year period. While the overview of these studies is somewhat useful, junior faculty members may find the learner assessment methods discussed in the review most interesting. Some studies used an OSTE, which is a direct observation of the participant’s teaching behaviors as determined by observable criteria. Other studies used videotaped teaching sessions as a means to directly observe participant’s teaching behaviors. Less rigorous assessment methods included teaching evaluations of the participants by their learners and participant self-assessment. Junior faculty members who design RAT programs should consider rigorous methods (direct observation) when assessing their learners or evaluating their program.

The second important point for junior faculty is the importance of the One-Minute Preceptor model as content for RAT programs. The One-Minute Preceptor model was the most commonly studied intervention; the authors state that the model is effective in increasing participants’ teaching ability, but do not provide specific evidence to support this.

Considerations for Faculty Developers

The article is a “lay of the land” summary for faculty developers. Aside from brief article summaries, faculty developers may extract the importance of the one-minute preceptor model as content for RAT or faculty development programs. Faculty developers may also consider OSTEs or videotaped teaching sessions as a means of providing summative and formative feedback to junior faculty or faculty members with sub-optimal teaching evaluations.

5. Bree KK, Whicker SA, Fromme HB, Paik S, Greenberg L. Residents-as-teachers publications: What can programs learn from the literature when starting a new or refining an established curriculum? J Grad Med Educ. 2014;6:237-248 [8].

Summary

The authors perform a systematic review of previously published RAT programs, focusing on each program’s reproducibility and quality of educational outcomes. A table includes 39 RAT programs from various medical specialties. Each study is evaluated according to whether the RAT program is reproducible, i.e. included a clear study design, goals and objectives, intervention duration and assessment, and lesson plans and materials. Studies are also evaluated for the rigor of educational outcomes. Using Kirkpatrick’s model [47] as a framework, each study is classified according to the level of outcomes achieved by the learners (Kirkpatrick score). Only one-quarter of studies measured outcomes at the fourth Kirkpatrick level (results).

Relevance to Junior Faculty Members

It may be difficult for faculty to implement previously published RAT curricula because important program details are often omitted or the programs do not have clear educational outcomes. This review provides a reference for faculty who are interested in using or modifying existing curricula to develop a RAT program at their institution. The article scores each published program on its reproducibility and level of educational outcome (Kirkpatrick score). Junior faculty are therefore able to identify programs that can be easily implemented and have measurable outcomes. Junior faculty may also extract important lessons about study design from this article, specifically the importance of striving for results-based outcomes in medical education research.

Considerations for Faculty Developers

Faculty developers should acknowledge that while the study is classified as a systematic review, the authors do not report adherence to Preferred Reporting Items for Systematic Reviews and Meta-Analyses (PRISMA) guidelines [49]. Regardless, the article provides an analysis of several published RAT curricula, including their reproducibility and outcomes. Since there is considerable overlap between faculty development programs and RAT programs, faculty developers may find this article to be a helpful review of existing RAT literature. Furthermore, the article may help faculty developers to guide junior faculty in RAT curriculum development and study design. It is notable that only 25% of the articles had educational outcomes at the fourth level of Kirkpatrick’s evaluation model, which indicates the need for high-quality curricula that provide educational outcomes at all levels of evaluation.

Limitations

Our literature search, while comprehensive, was not designed to be exhaustive. It is possible that relevant papers exist which were not included in our review. However, we sought the services of a medical librarian to assist in the completion of our literature review to provide a broad list from which to build our Delphi process.

In an effort to maintain a list of articles for initial review that was of a manageable size as well as relevance to today’s educators, we limited our articles to those published in the last 10 years. We believe that focusing on more recent articles will better reflect the challenges and limitations faced in the current medical education climate. There may have been pertinent articles that were published more than 10 years ago which were not reviewed.

In addition, every institution is unique. The success of a specific RAT program at one institution does not guarantee success when implemented at another institution. Faculty are encouraged to view the selected papers through the lens of their own education landscape to determine which techniques and programs will be most successful.

## Conclusions

The five articles identified in this paper can provide guidance and structure for junior and senior faculty members involved in planning or evaluating RAT curricula. Each paper has strengths for both junior faculty educators and faculty developers, and this list can be used as a guide when developing RAT programs to ensure a thorough approach that utilizes advanced educational strategies and evaluation techniques.
